# An Agent-Based Model of the Response to Angioplasty and Bare-Metal Stent Deployment in an Atherosclerotic Blood Vessel

**DOI:** 10.1371/journal.pone.0094411

**Published:** 2014-04-14

**Authors:** Antonia E. Curtin, Leming Zhou

**Affiliations:** 1 Department of Bioengineering, University of Pittsburgh, Pittsburgh, Pennsylvania, United States of America; 2 Department of Health Information Management, University of Pittsburgh, Pittsburgh, Pennsylvania, United States of America; University of Arizona, United States of America

## Abstract

**Purpose:**

While animal models are widely used to investigate the development of restenosis in blood vessels following an intervention, computational models offer another means for investigating this phenomenon. A computational model of the response of a treated vessel would allow investigators to assess the effects of altering certain vessel- and stent-related variables. The authors aimed to develop a novel computational model of restenosis development following an angioplasty and bare-metal stent implantation in an atherosclerotic vessel using agent-based modeling techniques. The presented model is intended to demonstrate the body’s response to the intervention and to explore how different vessel geometries or stent arrangements may affect restenosis development.

**Methods:**

The model was created on a two-dimensional grid space. It utilizes the post-procedural vessel lumen diameter and stent information as its input parameters. The simulation starting point of the model is an atherosclerotic vessel after an angioplasty and stent implantation procedure. The model subsequently generates the final lumen diameter, percent change in lumen cross-sectional area, time to lumen diameter stabilization, and local concentrations of inflammatory cytokines upon simulation completion. Simulation results were directly compared with the results from serial imaging studies and cytokine levels studies in atherosclerotic patients from the relevant literature.

**Results:**

The final lumen diameter results were all within one standard deviation of the mean lumen diameters reported in the comparison studies. The overlapping-stent simulations yielded results that matched published trends. The cytokine levels remained within the range of physiological levels throughout the simulations.

**Conclusion:**

We developed a novel computational model that successfully simulated the development of restenosis in a blood vessel following an angioplasty and bare-metal stent deployment based on the characteristics of the vessel cross-section and stent. A further development of this model could ultimately be used as a predictive tool to depict patient outcomes and inform treatment options.

## Introduction

In an atherosclerotic blood vessel, blood flow is restricted by the accumulation of plaque, which causes the walls of the vessel to become inflamed [1]. The subsequent narrowing of the lumen of the blood vessel by the plaque causes ischemia, and vascular intervention is usually required to compress the plaque and regain the lumen area to restore blood flow [2]. According to a report published recently, an estimated 492,000 patients underwent percutaneous coronary intervention (PCI) procedures in 2010 in the United States [3], and stents (drug-eluting stents and bare-metal stents) were deployed in 454,000 of these patients (or roughly 92% of all patients) during these PCI procedures [3]. Although the goal of a PCI intervention is to re-expand the lumen of the target blood vessel, the body’s natural wound healing response at the site of the intervention can cause a re-narrowing of the treated vessel, or restenosis, which often counteracts what would be an otherwise successful intervention [2], [4]. Up to 60% of such PCI and similar interventions to treat ischemic lesions fail because of restenosis [2], [5]. The ensuing target lesion revascularization caused by in-stent restenosis can be severe and detrimental to a patient’s recovery [6]. Some studies have shown that as many as one-third of patients with in-stent restenosis developed subsequent myocardial infarctions or unstable angina that required the patient to be hospitalized [7].

Animal models such as rats, mice, rabbits, and pigs have been used extensively to investigate the progression of restenosis in stented arteries and have provided a wealth of insightful information about this complication in the past several decades [8], [9]. Nevertheless, because computational models are useful for simulating situations that cannot be created in an animal and permit fast and precise perturbations of the simulation environment, they are conducive to identifying the major effectors of the process being simulated and present a viable alternative to animal models. Agent-based modeling is a computational modeling technique for simulating the actions and interactions of agents (such as cytokines, cells, tissues, and organs) in an environment of interest [10]. When agents interact with each other stochastically, their aggregate behavior leads to complex, emergent phenomena that represent the system as a whole. Agent-based models can provide both numerical values and overall *visual patterns* in the course of the simulations, which are typically very informative. The model presented in this article was developed with the NetLogo platform [11].

The rest of this article is broken into four sections: materials and methods, results, discussion, and conclusions. The materials and methods section presents the clinical events simulated, model construction procedure, model interface, outputs, parameter sensitivity analysis, and model validation procedures. The results section describes the results from various simulations for a single stent and overlapping stents, including quantitative values and visual outputs. The discussion section provides detailed explanation of the obtained results, limitations of the model, and plans for further development of the model. The conclusion section includes a brief summary of the model results and possible future directions.

## Materials and Methods

### A. Clinical Events Simulated

A normal coronary artery consists of three concentric layers, from the innermost to the outermost, called the intima, media, and adventitia. The intima is covered by a single layer of endothelial cells (**ECs**); the media comprises mainly smooth muscle cells (**SMCs**) and elastic tissues; and the adventitia is rich in fibroblasts and connective tissues [1], [2]. In the course of an inflammatory reaction, **platelets**, **neutrophils**, and **macrophages** are activated and recruited to the wound site. These cell types can produce pro-inflammatory or anti-inflammatory cytokines, such as **TNF-α** (tumor necrosis factor alpha) and **TGF-β** (transforming growth factor beta) [1], [12], [13]. After an angioplasty and subsequent stent deployment procedure, **stent struts** become part of the artery and are therefore included in this model from the beginning of simulation. These are the major contributors to restenosis development and are represented by agents in this agent-based model.

In addition to these major components, an atherosclerotic coronary artery (the blood vessel simulated in this model) has a lipid-rich core called plaque [14] between the intimal and medial layers. At the interface of the intima and the plaque, SMCs and elastic tissues may also be present. Depending on the thickness and composition of this particular layer (sometimes referred to as a “fibrous cap” in the literature), the plaque can be stable or vulnerable to breaking (*plaque rupture*) during the angioplasty and stent-deployment procedure. Plaque rupture can lead to an intense inflammatory reactions and be accompanied by an acute thrombosis, and therefore requires a different therapeutic approach for controlling acute inflammatory reactions and thrombosis, such as administering anti-platelet drugs and anti-inflammatory drugs [15]–[19]. The behavior of cells that contribute to restenosis development is altered in the presence of such drugs and is dependent on drug type and dosage. Because the goal of the model is to elucidate how the cell types and cytokines of interest contribute to in-stent restenosis development when drugs are not present, it is assumed that there is no plaque rupture during the angioplasty and stent procedure. The model solely simulates the vessel’s and body’s response to the injuries created by the angioplasty and stent procedure.

As a result, the processes simulated in the model are quite similar to the typical wound healing following tissue injury [20]. The first phase of the wound healing response involves the aggregation of platelets and the infiltration of inflammatory cells (such as macrophages and neutrophils), followed by proliferation and apoptosis of ECs and SMCs under the stimulation of inflammatory cytokines (such as TGF-β and TNF-α) [21]–[23]. Inter-patient differences in the extent and rate of wound healing are largely due to differences in physiological variables such as the cytokine-dependent SMCs and ECs proliferation rates.

**Figure 1:**
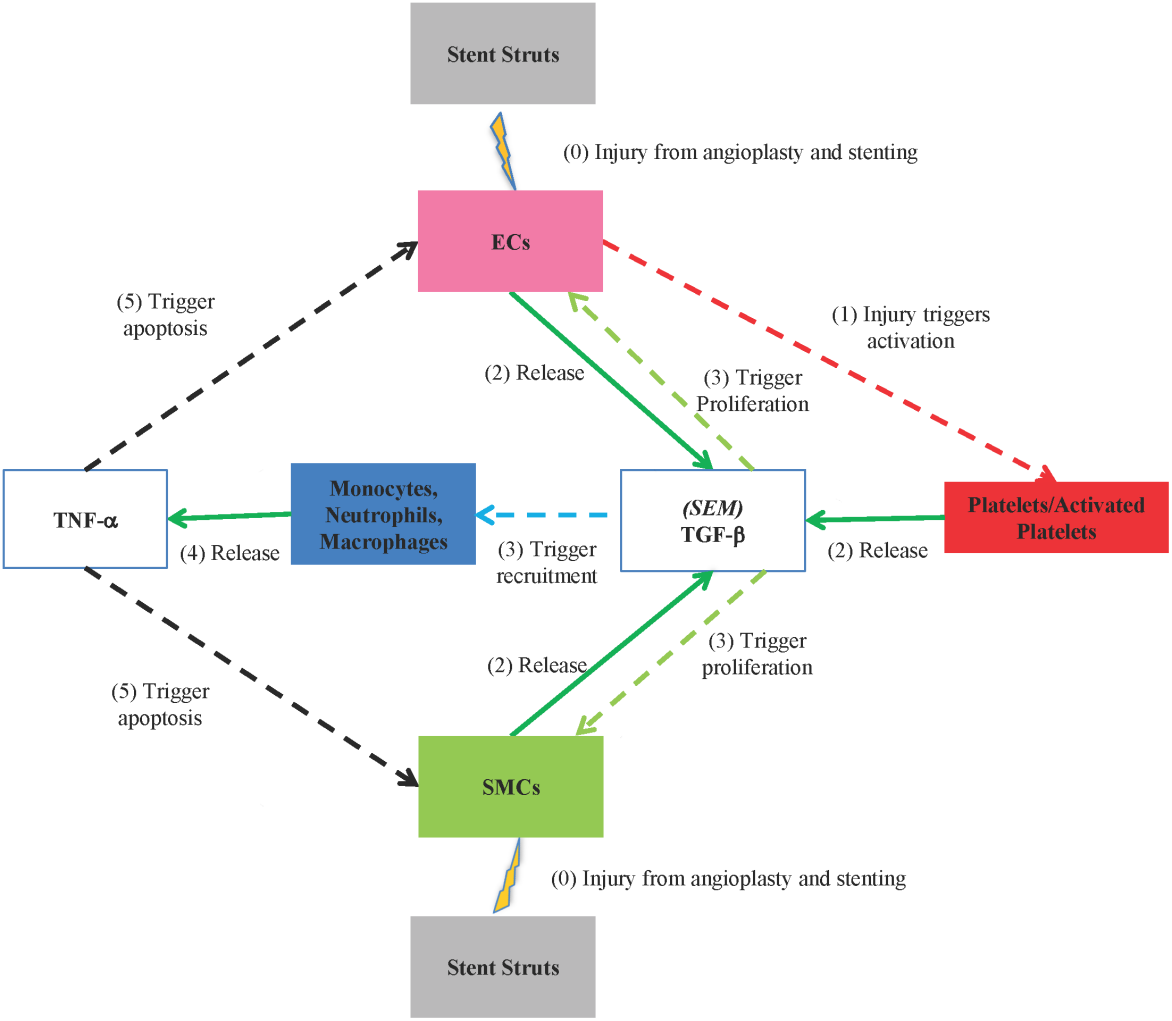
An interaction diagram for cells and cytokines represented in the model. The major players in the interaction are shown in boxes with colors. These colors are the same as the ones used in the visual output of the agent-based model. The solid green lines mean something is releasing and the dashed lines mean an event is being triggered. Here is the color code for events: light-green for proliferation, light-blue for recruitment, red for activation, and black for apoptosis. The numbers in the diagram indicate the order of those events at the beginning of a model simulation.

A holistic summary of the processes simulated in the model is presented below. Prior to simulation initiation, SMCs and ECs are injured during the angioplasty and stent procedure. The result of this procedure is the starting point of the model simulation. **Step 1**: Exposure of the subendothelial matrix (SEM) and collagens as a result of the endothelial cell damage immediately activates circulating latent platelets [24]. Activated platelets aggregate to form a growing thrombus and activate circulating latent platelets upon contact [24]. **Step 2**: SMCs, ECs, and activated platelets release anti-inflammatory cytokines such as TGF-β [25], [26]. **Step 3**: Sufficient amounts of TGF-β trigger the recruitment of monocytes and neutrophils to the wound site [27], [28]. *In vivo*, this recruitment is actually performed by the SEM, which releases TGF-β and other chemoattractants to draw monocytes and neutrophils to the wound site [29]–[31]. Monocytes migrate through the thrombus [30] and mature into macrophages when they reach the tissue [26], [32]. Neutrophils form contact-based aggregates with activated platelets and become integrated into the thrombus [30], [31], [33]. **Step 4**: Monocytes, neutrophils, and macrophages release pro-inflammatory cytokines such as TNF-α [34]. **Step 5**: Both pro- and anti-inflammatory cytokines (TNF-α and TGF-β) diffuse through the tissues adjacent to the site of their initial release. When the local concentration of TGF-β reaches a certain threshold, SMC and EC proliferation in that area will be triggered [35], [36]. When the local concentration of TNF-α reaches a given threshold, SMCs and ECs apoptosis will be triggered [37]. This simplified description of the *in vivo* interactions among the major cell types and cytokines of interest in the development of restenosis is illustrated in Figure 1. In this figure, these cell types and cytokines are color-coded to make it consistent with the colors of agents used in the model.

It is important to note that the *in vivo* interactions are much more complicated than this simplified description. For instance, TGF-β can act as both a pro- and anti-inflammatory cytokine at different stages of restenosis development [38], [39]. There are also many other cytokines and growth factors (e.g., interleukin 8, interleukin 6, platelet-derived growth factor, fibroblast growth factor, vascular endothelial growth factor, etc.) involved in the whole process [40]. In this model, TGF-β and TNF-α were used to represent anti-inflammatory cytokines and pro-inflammatory cytokines, respectively. The use of only a few cytokines in agent-based models of inflammatory-based healing responses is a frequently applied practice [41], [42]. This simplification is necessary because the precise roles of these cytokines within the complex *in vivo* environment are not clear while the presence of anti-inflammatory and pro-inflammatory cytokines are vital to the dynamic interactions among the major contributors to restenosis development [5], [40]. The major purpose of this work is to simulate the dynamic interactions among these major contributors and to determine their roles in the development of restenosis. Without proper simplification, this model with detailed interactions among a large number of cytokines and growth factors would not produce any meaningful results.

### B. Model Construction

#### i) Settings of the model

Corresponding to the structure of the coronary artery, the agent-based model developed in this work contains four major compartments in a simulated arterial cross-section: the **lumen**, **intima** or **neointima** layer (endothelial cells and some smooth muscle cells), the **media** layer (mainly smooth muscle cells), and the **adventitia** layer. The model also includes agents representing platelets, monocytes, neutrophils, and macrophages, along with cytokines such as TNF-α and TGF-β.

The environment of interest in the model is the treated blood vessel post-implantation of the stent. The simulated environment is a two-dimensional cross-section of the treated blood vessel. Although three-dimensional agent-based models can also be created, the use of a two-dimensional model is supported by the prevalence of the use of two-dimensional cross-sections in assessing vulnerable atherosclerotic plaques [43]. The cross-section with the smallest lumen diameter is the limiting factor for blood flow and is thus the case of greatest interest. The simulated results from this two-dimensional model can also be directly compared with those obtained in published serial angiography or ultrasound studies.

The agent-based model is created on a 150×150 grid, which corresponds to 22,500 square patches in total. All the agents in the model are contained on these patches in the grid. In the NetLogo program, one distance unit is defined as the distance between the centers of two adjacent patches in the grid, which is also the side length of a patch. In this model, one distance unit represents 0.05 mm *in vivo*. The size of a patch is also used as a reference for agent sizes. For instance, if the size of an agent is 1.0 unit, the agent’s diameter (for a circular agent) or side length (for a square agent) is the same as that of a patch side.

In this model, one time step represents 6 hours. In the agent-based model, each agent’s behavior follows a list of *behavioral rules* (Table 1). These behavioral rules are implemented as functions in the model within the NetLogo platform. When the agent-based model is running, in each time step the model checks the agents’ current situation and determines which function(s) should be executed and what input values should be utilized. If the current value of a variable is needed by a function, the model assesses the corresponding local environment to determine the correct value to use. Here an agent’s *local environment* is defined as the patch with the desired agent on it, and its 8 immediately adjacent patches in the grid space (illustrated in Figure S1 in Text S1). For instance, if a function requires the local TGF-β concentration, the model will count the number of TGF-β agents on the patches in the local environment and calculate the concentration value accordingly (counts/volume).

**Table 1:** A list of simplified rules of agents in the model.

Agent	Interaction rules
Platelets	Latent platelets move randomly in the lumen.
	When latent platelets meet injury sites or activated platelets, they are activated.
	Activated platelets aggregate together and lead to thrombus formation.
	Platelets die when they reach their given life-span.
TGF-β	Representing anti-inflammatory cytokines
	Activated platelets, SMCs, and ECs release TGF-β.
	A greater injury site triggers an increased release of TGF-β.
	A change in lumen area affects the release rate of TGF-β.
	Recruiting monocytes and neutrophils
	If the local concentration of TGF-β goes beyond a threshold, SMCs and ECs proliferation will be triggered.
TNF-α	Representing pro-inflammatory cytokines
	Monocytes, neutrophils, and macrophages release TNF-α in the same rate, and the release is triggered by a TGF-β gradient.
	If the local concentration of TNF-α goes beyond a threshold, SMCs and ECs apoptosis will be triggered.
Monocytes, neutrophils,macrophages	When the local TGF-β concentration in the intimal layer exceeds the femtomolar level, circulating neutrophil and monocytes appear in the lumen.
	The migration rate of these monocytes, neutrophils, and macrophages is 1μm/min. During the migration, if neutrophils meet activated platelets, neutrophils attach to the thrombus.
	Monocytes move through the thrombus and change into mature macrophages.
	Some macrophages stay with stent struts and simulate a foreign body response. Other macrophages are elsewhere and trigger typical inflammatory actions.
SMCs	Injured intimal SMCs replicate twice every proliferation cycle.
	Proliferating medial SMCs can migrate to the intimal layer and change to intimal SMCs.
	Each patch in the grid can only hold 15 SMC agents. Once this limit is reached, the extra SMCs are pushed to neighborhood patches in the direction of the lumen.
ECs	ECs always reside on patches adjacent and interior to the forward-most patches holding SMCs. When SMCs move toward the lumen, ECs move accordingly.

An agent-based model is advantageous in that the number of each type of agent can be tracked continuously in one run of a simulation. All these numbers of agents in different types are discrete values obtained by counting each type of agent on all the patches in the grid. In some cases, the numbers of agents themselves are sufficient for providing the desired information, for instance, the populations of ECs and SMCs. In other cases, these numbers of agents need to be compared with values reported in the literature, for instance, the concentrations of TNF-α and TGF-β. In the latter situation, the number of agents in the model collected during a simulation is converted to a value with the same unit as the ones measured in experiments. This conversion mechanism is set up when the model is initially constructed and is not changed in any subsequent simulations.

In the model, the coronary artery cross-section environment is populated by multiple types of agents. Some of these agents are initially bound to a specific location in the grid space and some of them freely circulate in the lumen. The agents initially assigned to a specific location are based on the *in vivo* makeup of the vessel wall layers. The intimal layer is chiefly populated by a small number of SMC agents and EC agents, representing the endothelial monolayer. The medial and adventitial layers are populated entirely by SMC agents. The other agent types present in the model prior to simulation initiation are platelet agents, latent TGF-β agents, plaque agents, and stent strut agents. Stent strut agents (representing the struts in a bare-metal stent) are evenly placed around the initial lumen boundary and remain unchanged during the course of a simulation. Illustrations from Thim *et al*. were consulted during the creation of the visual environment of the model prior to the start of a simulation [14]. The plaque size and location are set prior to simulation initiation and are maintained throughout the simulation. Once a simulation starts, other types of agents, such as monocytes, neutrophils, macrophages, appear in the model in accordance with the behavioral rules described in the following section.

#### ii) Behavioral rules of agents in the model

In an agent-based model, the behaviors of agents are controlled by type-specific rules. In this model, the agents’ behavioral rules control replication rates, responses to the presence of other agents, and interactions of agents with their environment. In certain cases, the type of agent can be changed and consequently, the corresponding behavioral rules of the new type would be applied to the agent. This transition is typically based on the agent’s position, type, and the local environment. One example of an agent type reassignment is the transformation from latent platelets to activated platelets. When circulating latent platelets encounter the wound site, their agent type is changed to activated platelet to reflect the activation event that would take place *in vivo*. In the model, latent platelets and activated platelets are distinct agent types and they are associated with different colors, sizes, and behavioral rules.

The behavioral rules for agents in the model are compiled according to an extensive literature survey and are summarized in a list form in Table 1. These rules have been simplified in order that they may be presented in the table. Further explanation of these rules and the corresponding supporting evidence are provided in Text S1.

#### iii) Parameters

The second main component of an agent-based model is the parameters associated with different types of agents. In this model, the parameters that are associated with the different cell types were set to the correct relative proportions based on data from the literature. Each agent is associated with multiple parameters that govern its behavior. The size and color of each agent were selected so that the simulation output is similar to a real situation and easy to understand. The parameters for each agent type in the model are presented in Table 2. Further details about how these parameters were chosen and implemented in the model are explained in Text S1.

**Table 2:** Summary of parameter values governing release rates, cytokine sensitivities, migration rates, proliferation rates, color, size, and lifespans of agents in the model.

Agent (color, size)	Parameter	Parameter Value of Reference	Source
TGF-β (invisible, 0.0002)	Release rate	0.02 pg/10 hours/cell	[74]
TNF-α (invisible, 0.0002)	Release rate	0.02 pg/10 hours/cell	[74]
SMCs (green, 0.6)	TGF-β sensitivity	3 ng/ml	[49]
	Migration rate	20 μm/hour	[75]
	Proliferation rate	Population doubles every 30 hours	[76]
Monocytes, neutrophils, and macrophages	TGF-β sensitivity	1 fmole	[28]
ECs (pink, 0.42) and SMCs	TNF-α sensitivity	4 ng/ml	[37]
	Maximum apoptosis rate following TNF-α exposure	15% of the population	[77]
Platelets (red, activated: 0.4, latent: 0.3)	Lifespan	5–10 days	[78]
Monocytes (blue, 0.54)	Migration rate	1 μm/min	[79]
	Lifespan	3 days	[80]
Macrophages (cyan, 0.54)	Lifespan	45 days	[81]
Neutrophils (light blue, 0.54)	Lifespan	5 days	[82]
Stent struts (gray, 2.7)	Default size	0.1 mm	[44]
	Default number of struts per stent	20	[45], [46]
Plaque (violet, 0.7)	Lifespan	No limit	

### C. Model Interface, Outputs, and Parameter Sensitivity

#### i) Modifiable parameters in the model interface

The model interface consists of modifiable parameters and quantitative and visual outputs. These modifiable input parameters include the lumen diameter (in mm) following angioplasty and stent implantation, the stent length (in mm), the number of stents, and the arrangement of the stent struts in the vessel cross-section. All of these parameters have default values. Prior to the initiation of the model in each simulation, these parameters can be set to desired values according to the purpose of the simulation.

Some input parameters were incorporated into the model due to the increasing interest in overlapping-stent cases and the effects of novel stent designs. Parameters specifically relevant to these topics are the number of stents in the simulated cross-section, the number of struts per stent, and the spacing of the stent struts, all of which can be manipulated in the model interface. When simulating an overlapping-stent scenario, the same input parameters are applied to both stents. In the model interface, there are two options for the two stent strut arrangement: aligned and staggered. In the aligned arrangement, the struts of the interior stent are placed right above the ones of the exterior stent. In the staggered placement, each of the struts on the interior stent is placed at the center of two adjacent struts on the exterior stent.

For both single- and multi-stent simulations, the size of the stent struts and either the number of stent struts or the spacing between two adjacent struts (in mm) can be set at the beginning of the simulations. The default stent strut size is 0.1 mm [44], and the default number of struts per stent is 20 in the vessel cross section [45], [46]. For any designated number of struts in the stent(s), those struts are always evenly spaced around the post-stenting boundary of the lumen. For any given spacing between two adjacent struts, an appropriate number of struts is determined by dividing the post-stenting lumen circumference by the selected spacing and rounding to the nearest integer value.

An additional modifiable parameter is the vessel wall thickness. This parameter does not appear in the model interface, but its value is calculated based on the initial lumen diameter. Since the vessel thickness is typically not reported in experimental studies, in this model the default wall thickness is defined as being equal to the given lumen radius, based on the observation that the typical ratio between the lumen radius and the vessel wall is roughly 1∶1 [47].

#### ii) Simulation outputs: value measurement protocols

The agent-based model can produce both numerical and visual outputs in the user interface. The quantitative outputs are the final lumen diameter, the days to reach a stable lumen diameter (the time to lumen diameter stabilization), the percent change in lumen area, and the local and serum concentrations of TGF-β and TNF-α (both in ng/ml, or nanogram/milliliter, or 10^−6 ^kg/m^3^). In this model, one of the visual outputs is a plot that tracks the population of SMC and EC agents during a simulation. The other visual output is a color-coordinated diagram of the blood vessel cross-section, which is continuously updated during each simulation to illustrate the positions of different types of agents in the model and the changes to the lumen area. In this model, a few variable measurement protocols were implemented to obtain the information for all these valuable outputs.

To measure the changes in the lumen cross-sectional area, the model used the location of intimal EC agents as the reference points for the boundary of the new lumen. This lumen diameter measurement method was utilized to ensure that the model monitors the worst-case lumen obstruction, which is considered to be most clinically significant. Consequently, percent change in lumen area was calculated according to the equation below:
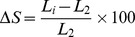
where 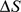 is the percent change in lumen area, 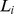 is the initial lumen area (in mm^2^) prior to the start of a simulation and 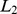 is the new lumen area (in mm^2^) based on the minimum lumen diameter (in mm).

In the numerical outputs of the model, the concentrations of TGF-β and TNF-α are reported as values comparable to the ones reported in the literature (in ng/ml). The local cytokine concentrations are calculated by counting the numbers of these cytokine agents on a patch of interest and its adjacent 8 patches (the local environment), and then dividing this count by the volume for the chosen patch set. For any patch of interest, the corresponding volume of the local environment is defined as V = C×AP, where V is the volume, C is a constant for the thickness of the patch, and AP is the area of 9 patches. In the numerical output in the model interface, the reported cytokine concentrations include both the local and serum concentrations of the cytokines. The local cytokine concentrations directly affect the proliferative and apoptotic behavior of those cell agents within the measurement perimeter according to the behavioral rules. The serum concentration of the cytokines does not affect the behavior of any cells in the model but rather serves as a checkpoint to ensure that the net cytokine release does not exceed normal physiological ranges. Acceptable serum TNF-α values were determined to be between 0 and 10 ng/ml, based on *in vivo* studies [37], and the same range was considered to be acceptable for the serum TGF-β concentrations [48], [49].

The model constantly reports the simulated days post-intervention in the model interface. In each simulation, the number of days from the beginning of the simulation to the end of TGF-β release is used as the lumen diameter stabilization time (in days). After this point, data are recorded for an additional 60 simulated days to confirm that the lumen diameter had become static.

#### iii) Simulation outputs: examples of visual lumen area changes

**Figure 2:**
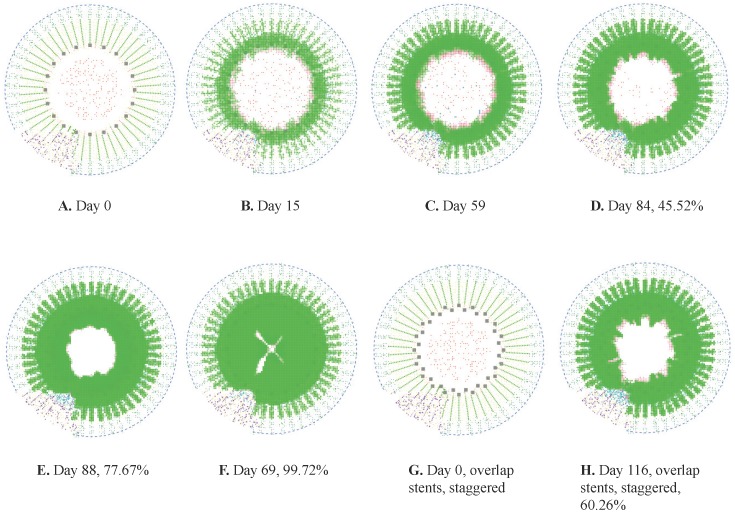
Visual outputs from single-stent and overlapping-stent simulations. Simulations utilized the initial vessel parameters from Kimura et al. [50]. 2A–2D: Snapshots from a single-stent simulation at different days (days 0, 15, 59, and 84). 2E–2F: Final results from two single-stent simulations with different levels of restenosis. 2G–2H: The initial and final images from one overlapping-stent simulation.

The visual outputs for the blood vessel cross-section from both single-stent and overlap-stent simulations using the initial parameters from [50] are shown in Figures 2A–2H. These visual outputs were randomly selected from simulations. Figures 2A–2F are the results from single-stent simulations while Figures 2G and 2H are the results from an overlapping-stent simulation. Figures 2A–2D are all from one individual simulation while Figures 2E–2G are from different simulations. Figure 2A illustrates the initial setting of the vessel cross-section right after the initiation of the simulation, which represents the status of the vessel immediately following the angioplasty and stent implantation procedure. Visible agents at this stage include platelets (red) in the lumen, stent struts (gray squares), ECs (pink), SMCs (green), and plaque (violet, bottom-left corner). Figure 2B presents the vessel cross-section 15 simulated days after the stent procedure. Monocytes (blue), neutrophils (light blue), and macrophages (cyan) were present in the model by this time point. The population of SMC agents (green) increased significantly during the 15 days. Figure 2C shows the status of the blood vessel 59 days after the procedure. Figure 2C clearly reflects the proliferation of EC and SMC agents and migration of SMC agents towards the lumen. Figure 2D illustrates the ultimate results of this simulation. The lumen diameter was stable after 84 simulated days and 45.52% of the original lumen area was occupied by the neointima. In this particular case, the neointima did become thicker, however, it did not reach the point of restenosis (greater than 50% lumen area loss [51]). In other simulations, the SMCs and ECs keep proliferating and migrating towards the lumen. The neointima may then occupy a large portion of the lumen (≥50% of the lumen area). This phenomenon is demonstrated in Figure 2E, which is the result from a different simulation from Figure 2D. The lumen was stabilized after 88 days and the neointima occupied 77.67% of the original lumen area. The result depicted in Figure 2F, which is from yet another separate simulation, represents the worst-case result. In this instance, the neointima advanced to such an extent that it occupied the entirety of the lumen space, which occurred 69 days after the intervention. In the cases of both Figures 2E and 2F, restenosis would have been developed in the simulated artery since the neointima occupied more than 50% of the original lumen area. Figures 2G and 2H display the initial setting and the final result of an overlapping-stent simulation, respectively. A staggered stent strut arrangement was selected for this simulation, which is illustrated in Figure 2G.

#### iv) Parameter sensitivity analysis

The purpose of the parameter sensitivity analysis was to identify any parameters for which a small change in the parameter value resulted in a *significant alteration* in the behavior of the model. Sometimes, the model’s sensitivity to a particular parameter does not have an obvious connection to *in vivo* events based on the literature. The sensitivity analysis was also meant to check the *reasonableness of the model results* when small perturbations were made to model parameters. Any physiologically impossible or implausible results stemming from minor alterations to model parameters would highlight a potential weakness in the model construction. As exhibited by the results of the sensitivity analysis, no such weaknesses were identified during the course of the testing on this model.

A few hundred simulations were performed to confirm the consistency of the simulation results. No irregularities (i.e., a simulation reaching completion within a timeframe of only a few simulated days, dramatic/unexpected changes of simulated behavior in the middle of a simulation, or failure to reach lumen diameter stabilization, etc.) were observed in these test simulations. For each given set of initial parameters, the final lumen diameters obtained from those simulations were consistent. A simple test was conducted using one set of input parameters to demonstrate the consistency of the results. The test procedure and results are presented in Text S1. This simple test indicates that the average values of two groups with the same input parameters and 10 random runs are highly consistent. Therefore, it is sufficient to have 10 runs per simulation for data reporting. In the rest of this article, all of the reported results are the average values of 10 random runs of the simulation with the indicated parameters.

The results of the sensitivity analysis for functions with direct physiological meaning are presented in Tables S1 and S2 in Text S1. After all simulations for one alteration were completed, the model was run in its normal configuration for two simulations to confirm all changes had been reversed prior to performing the next alteration. The results presented in Tables S1 and S2 in Text S1 indicate that the model is extremely sensitive to functions related to intimal and medial apoptosis and proliferation, inflammatory cell adherence and migration, and TNF-α. The influence of intimal and medial apoptosis and proliferation functions on the final results of the model is expected based on the *in vivo* pathogenesis of restenosis, and the significance of the inflammatory cells (monocytes, neutrophils, and macrophages in this model) is because they release cytokines in their local environment. The adventitial SMCs’ apoptotic and proliferative functions do not have a significant impact on model outcomes because adventitial SMCs do not migrate to other layers of the vessel wall and thus changes in the adventitial SMC population do not contribute to the development of neointima. As noted in Table S2 in Text S1, the endothelial proliferation functions do not contribute to increases in neointima thickness due to the single layer feature of ECs in the neointima layer. The locations of ECs are, however, crucial to the *measurement* of the lumen diameter.

### D. Model Validation and Overlapping-stent Simulation Procedures

The goal of the model validation testing was to assess the integrity of the model’s quantitative outputs. To this end, simulations were performed using vessel parameters from three serial imaging studies [50], [52], [53]. These three imaging studies were selected specifically because these studies reported both initial and final lumen diameters and had extended follow-up times and high participant retention rates. For each comparison, 10 runs of each simulation were performed with the parameters of the corresponding imaging study as the input parameters of the model. The average final lumen diameter and average time to lumen diameter stabilization (± standard deviation) from these 10 runs of each simulation were then reported. The time to lumen diameter stabilization was rounded to the nearest day. The outer diameters of the imaged vessels were not given in any of these three imaging studies, and therefore the default vessel thickness (the same as the given lumen radius) was used in these simulations.

The plaque in the model was placed at the bottom left corner of the simulated cross section across all simulations. Because the vessel’s cross-section is a circle, repositioning the plaque along the circumference should not affect the simulation results. The plaque size in the model was also preset at a specific value, described by four boundary values (x_1_, x_2_, y_1_, y_2_). The location and size of the plaque can be manipulated by modifying these four boundary values within the code of the model. However, because there was no information about the position and size of the plaque in the targeted vessels imaged in the comparison studies, the plaque location and size were maintained at the model’s default configuration. If the location and size of the plaque need to be changed, the new boundary of the plaque should not protrude into the lumen or distort the stent. This is required by the innate assumption that the blood vessel’s cross-section is a circle after the angioplasty and stenting procedure. In all other respects, the consequences of plaque rupture should be taken into account in the model. As indicated at the beginning of the Materials and Methods section, this model does not incorporate the contribution of a ruptured plaque to the inflammatory reactions in the process of restenosis development.

For the overlapping-stent simulations, the expected result was an increase in lumen cross-sectional area loss as a consequence of the increased vessel wall damage inflicted by the second set of stent struts and the corresponding increased intimal hyperplasia [10]. Overlapping-stent simulations were run using the initial post-implantation vessel parameters from Kimura et al. [50]. This study was chosen after the completion of the model validation simulations due to the closeness of the model’s results with the results reported in the comparison study. Ten runs of each overlapping-stent simulation were conducted to make sure that the obtained results were stable.

## Results

### A. Single-stent Simulations for Model Validation

#### i) Final lumen diameter

The average final lumen diameter and the average time to lumen diameter stabilization for each comparison simulation are given in Table 3. Across all three sets of validation simulations, the average values of the final lumen diameters generated by the model were all within one standard deviation of the means reported in the corresponding serial imaging studies from which the input model parameters were taken. The results from the simulation using the parameters from Chamié *et al*. differed more from the reported mean diameter than the results for the other two sets of simulations [52]. This discrepancy is likely due to the size of the vessels investigated in the Chamié *et al* study. Chamié *et al* specifically evaluated stent patency in smaller vessels. A smaller diameter atherosclerotic blood vessel may undergo stress-induced remodeling, resulting in a different lumen radius to vessel wall thickness ratio [54]. However, because no vessel wall thickness values were reported in the study by Chamié *et al*., the validation simulations were conducted with the default 1∶1 ratio between lumen radius and vessel wall thickness. If accurate values of vessel thickness were available, the accuracy of the model predictions would likely increase.

**Table 3:** Initial parameters and final results from serial imaging studies used for comparison by Kimura et al. [50], Hoffman et al. [53], and Chamié et al. [52] and model final lumen diameter and time-to-stabilization results.

Comparison Study	Measurement method	Study initial LD* (mm)	Study finalLD (mm)	Study follow-up time (months)	Model finalLD (mm)	Model time to LD stabilization (days)
Kimura et al.	Angiography	2.9±.04	2.2±.6	3–6	2.13±.02	89±7
Hoffman et al.	Intravascular ultrasound	3.19±.51	2.12±.82	5.4±3.8	2.34±.02	82±16
Chamié et al.	Intravascular ultrasound	2.39±.13	1.28±.74	7.2±1.0	1.75±.05	93±15

*LD = lumen diameter.

#### ii) Time to lumen diameter stabilization

The average time to lumen diameter stabilization from the model validation testing is also presented in Table 3. The time to lumen diameter stabilization generated by the model paralleled the study follow-up times for all three studies, in spite of the inter-study variation in follow-up periods. It is pertinent to note, however, that these investigator-determined follow-up periods may not reflect the actual time needed for lumen diameter stabilization *in vivo*.

#### iii) Percent change in lumen area

**Figure 3:**
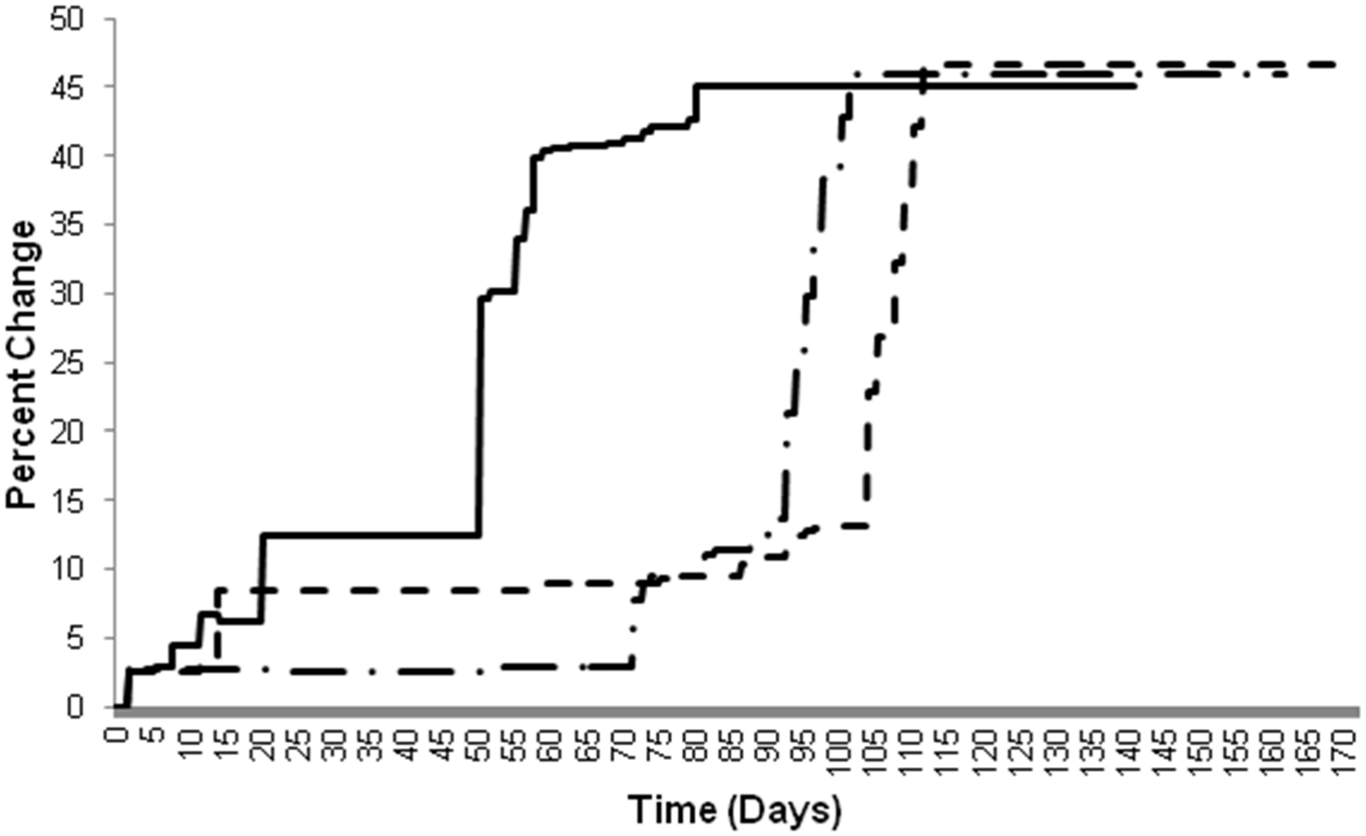
Percent change in lumen area in single-stent simulations from each set of validation simulations. Simulation using Kimura et al. parameters = solid; Hoffman et al. parameters = dash-dot; Chamié et al. parameters = dashed.

Due to wide variations in the method of calculation for percent change in lumen cross-sectional area reported in the literature, the values of percent change in lumen area from the model were not compared with the values reported in the literature. Instead, the average percent change in lumen area over time generated from model simulations was compared across the three sets of validation simulations. Figure 3 displays the simulated percent change in lumen area over time.

While the initial parameters, final lumen diameter, and time to lumen diameter stabilization varied according to input parameters, the curves in Figure 3 show that the percent change in lumen diameter followed the same trajectory across all three sets of simulations from simulation initiation to lumen diameter stabilization. The consistent nature of these curves reflects consistent cell and cytokine positive- and negative-feedback interactions across simulations and reinforces the validity of the numerical results presented in Table 3. If the path to lumen diameter stabilization varied significantly when different input parameters were used, it would indicate that the cell and cytokine interactions were not the main effectors of the lumen diameter change over time and the model results were artificially affected by the input parameters.

#### iv) Comparison of local cytokine levels

As discussed earlier in the Materials and Methods section, the serum cytokine concentration does not affect the behavior of any agents in the model. The serum concentrations of TGF-β and TNF-α serve as a checkpoint to ensure that the cytokine release does not exceed normal physiological ranges. As noted earlier, TGF-β and TNF-α concentrations are between 0 and 10 ng/ml. In all three sets of validation simulations, the serum cytokine concentrations remained within the normal range.

Local cytokine concentrations, however, are critically important to the functionality of the model as the local cytokine concentrations dictate the changes in the SMC and EC populations. For all validation simulations, the local cytokine levels for both TGF-β and TNF-α were within the expected ranges, which was the same as the normal serum range defined above. The maximum local TGF-β concentration experienced in the 30 runs of validation simulations was approximately 6 ng/ml, and the maximum local concentration for TNF-α was approximately 9 ng/ml.

### B. Overlapping-stent Simulations

The average final lumen diameter for the overlapping-stent simulations (using the Kimura *et al*. parameters [50]) was 1.79±.02 mm^2^, and the average time to lumen diameter stabilization was 112±8 days. These results represent a 14.5% decrease in average final lumen diameter and a 25.6% increase in the average time to lumen diameter stabilization from the single stent simulation results that used the same input parameters.

The overlapping-stent simulations exhibited an increase in lumen area loss and in time to lumen diameter stabilization. Both of these findings were expected based on the literature. The increased damage to the vessel wall caused by the additional stent resulted in increased apoptosis at the time of the intervention. The increased cytokine release also recruited more inflammatory cells, which released more TNF-α, requiring a prolonged vessel recovery time. The increased damage also triggered a chain reaction of increased cytokine release and subsequent increased cell proliferation and migration, which created amplified intimal hyperplasia. This reasonable explanation may indicate the qualitative expectation based on the literature, however, it cannot provide quantitative results when some specific input parameters are given. The simulations conducted in this agent-based model can provide the desired quantitative values.

### C. Comparison between Single-stent and Overlapping-stent Simulation Outcomes

#### i) Percent change in lumen area

**Figure 4:**
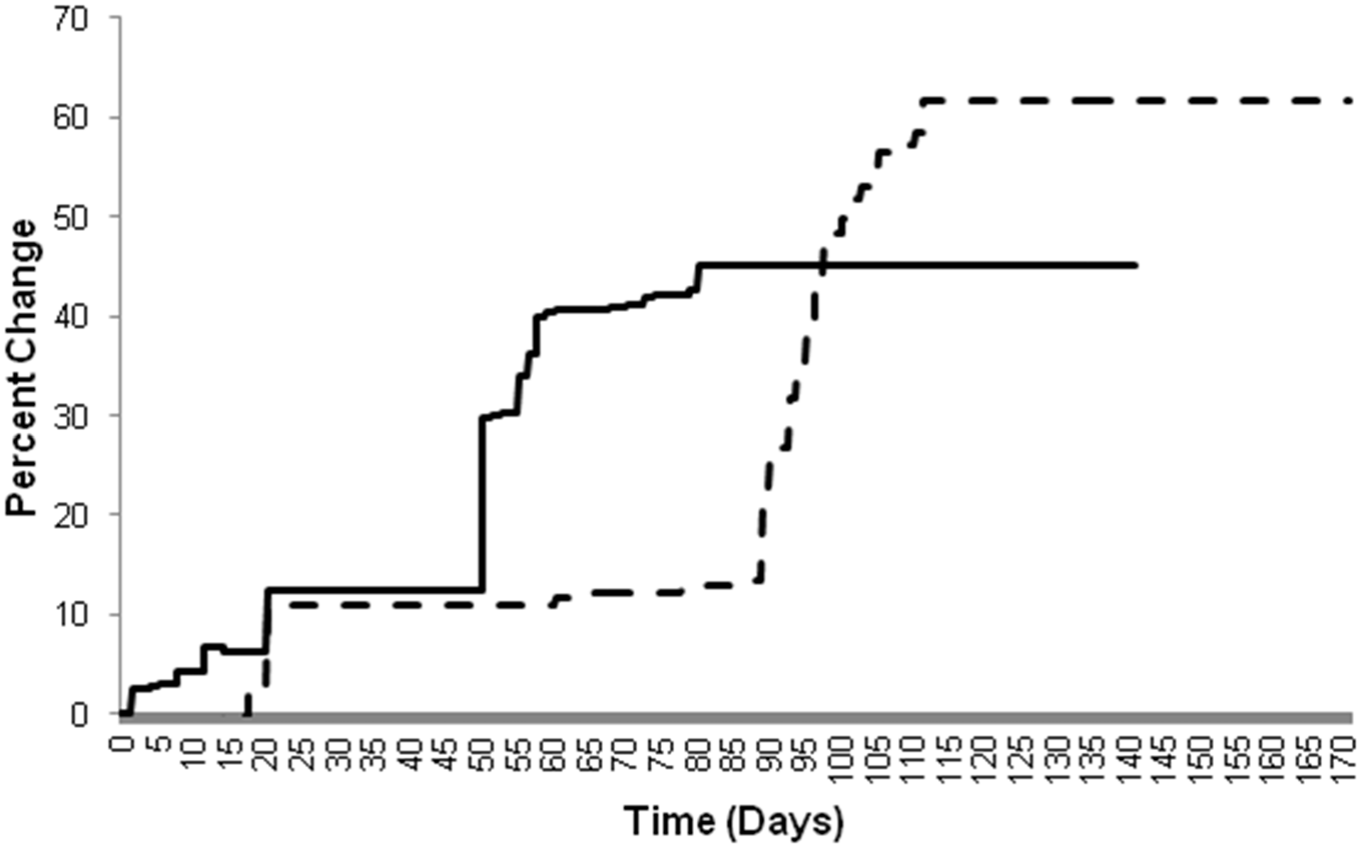
Comparison of percent change in lumen diameter in single-stent and overlapping-stent simulations. Single-stent simulations = solid and overlapping-stent simulations = dashed, all simulations using parameters from Kimura et al. [50].

Figure 4 presents the average percent change in lumen area over time from the single-stent simulations (solid line) and the overlapping-stent simulations (dashed line). Both sets of simulations utilized the initial parameters from Kimura *et al*. [50]. The differences between the two curves in Figure 4 illustrate the effects of altered hyperplasia-apoptosis balance. Although the trajectory of the overlapping-stent simulation curve is similar to the results for the single-stent simulations in Figure 3, the curve of the overlapping-stent simulation (dashed line) exhibits a phase delay with respect to the single-stent case (solid line). This delay is likely due to the increase in initial levels of apoptotic cell death, caused by the presence of the second stent. The lower initial SMC population in the case of overlapping-stent likely contributed to the delayed re-growth of the intima. However, the increased vessel wall injury also triggered an increase in the number of cells releasing the anti-inflammatory cytokine TGF-β. Thus, once the concentration of TGF-β reached a certain level, the change in lumen diameter was accelerated with respect to that in the single-stent simulation. This is reflected in the slope and magnitude of the curve for overlapping-stent simulations following the phase delay.

#### ii) Local cytokine concentrations

Overall, the concentrations of local TNF-α and TGF-β were higher in the overlapping-stent simulations than in the single-stent simulations. While the TNF-α levels were only higher by approximately 1 ng/ml, the increase in the TGF-β levels was more significant, >2 ng/ml, which represents a 33% increase. Notably, neither increase in local cytokine concentrations elevated the total concentrations beyond the physiologically appropriate limit (≤10 ng/ml).

The increased local cytokine concentrations seen in the overlapping-stent simulations provide support for the proposed mechanisms behind the differences between the single- and overlapping-stent simulations. Specifically, the altered local cytokine levels coincide with the changes seen in the lumen diameter data and in the percent change in lumen diameter curve. The increased TGF-β levels are a reflection of the increased hyperplasia, and the increased TNF-α levels are due to the increase in the number of recruited inflammatory cells (monocytes, neutrophils, and macrophages) drawn to the wound sites by the increased TGF-β levels.

#### iii) Cell population changes

**Figure 5:**
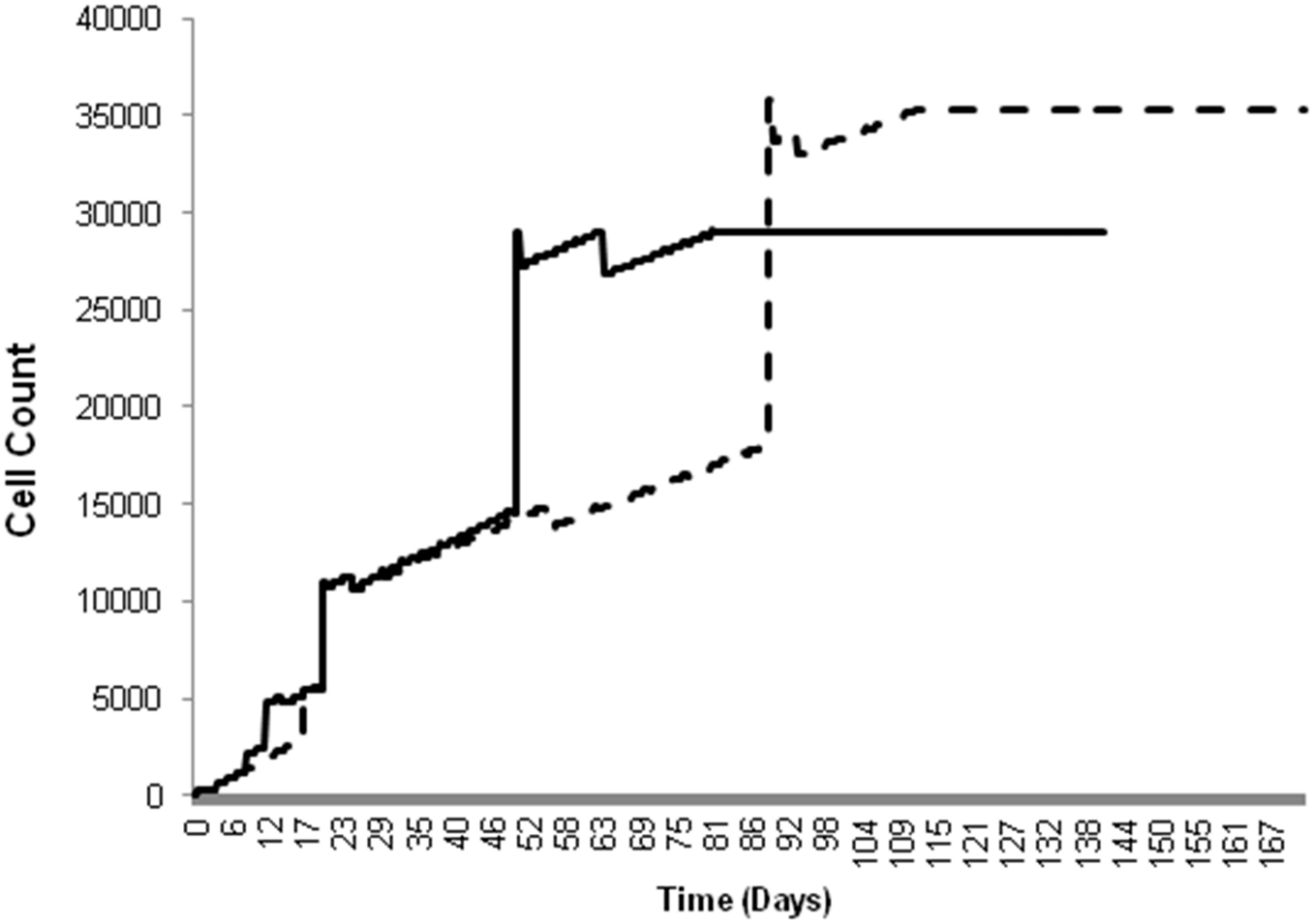
Comparison of the time course of SMC population changes in single-stent and overlapping-stent simulations. Single-stent simulations = solid and overlapping-stent simulations = dashed, all simulations using parameters from Kimura et al. [50].

**Figure 6:**
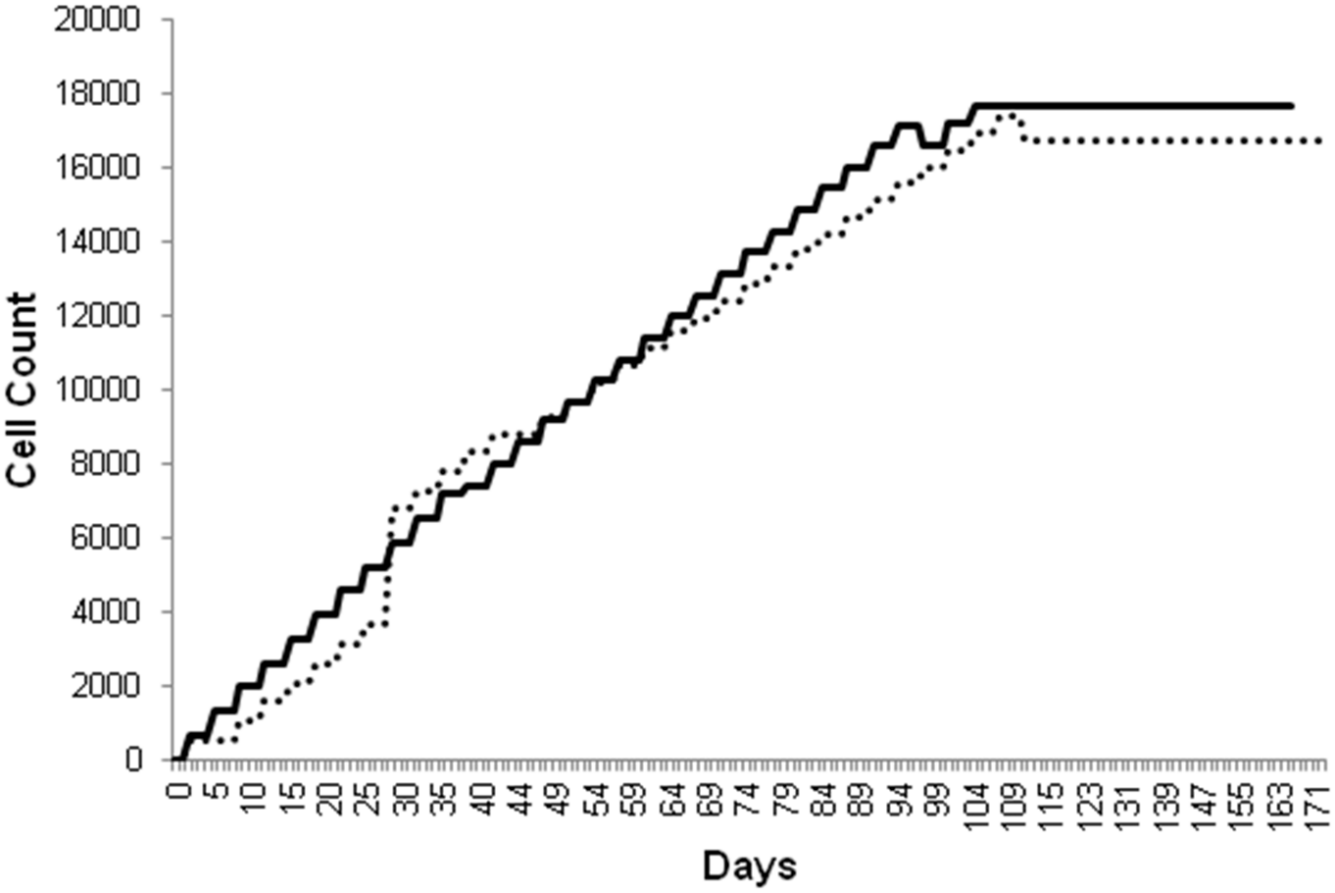
Comparison of the time course of EC population changes in single-stent and overlapping-stent simulations. Single-stent simulations = solid and overlapping-stent simulations = dashed, all simulations using parameters from Kimura et al. [50].

One set of results for the percent change in the SMC population and the EC population over time from the single-stent and overlapping-stent simulations utilizing the initial parameters from Kimura *et al*. [50] are plotted in Figures 5 and 6, respectively.

The effects of the altered cytokine concentrations in the overlapping-stent simulations are reflected mainly in the SMC population. The SMC population curve for the overlapping-stent simulation displays the same phase delay coupled with a slope and magnitude increase seen in the percent change in lumen diameter graph for the overlapping-stent simulations.

In contrast, the effects of the altered cytokine concentrations on the EC population appear to predominantly affect the initial population and did not significantly alter the rate or magnitude of the subsequent population growth. This disparity in the behavior of the SMC and EC populations is likely due to the reduced aggregate population of ECs in the model compared to SMCs. There are more than twice as many SMCs as there are ECs present in the model by the time of lumen diameter stabilization in a number of simulations, and the effects of the altered cytokine levels were more readily apparent on the larger scale.

## Discussion

By utilizing the developed behavioral rules and the vessel’s post-intervention parameters, the agent-based model described in this article successfully simulated the response of an atherosclerotic blood vessel to an angioplasty and bare-metal stent deployment procedure as described in the literature. In addition, the model was capable of accurately simulating the effects of overlapping-stent deployment.

Simulations with three sets of parameters from imaging studies verified the correctness and robustness of the model. The reported results indicate that the model can reproduce the results observed in the serial imaging studies by using the input parameters obtained from these studies. Based on these results, it is obvious that this model can be used to perform simulations with different input parameters and produce correct predictions without any alterations to the model’s mechanisms. The reasonableness of the percent change in lumen area and time for lumen stabilization results obtained from these simulations clearly demonstrate the accuracy of the model.

The results from the overlapping-stent simulations can also be used as a prediction of the *in vivo* response to multi-stent deployment. In a clinical setting, rather than in experiments, two overlapping stents would not be placed concurrently. The second stent is usually implanted after the restenosis in the first stent has developed to a serious stage. This is one limitation of the model in overlapping-stent simulations.

In the simulation for lumen area change in the case of a single stent implantation, the simulated time to lumen diameter stabilization was similar across all three compared imaging studies (Table 3). However, since the follow-up periods for the study were predetermined by the respective investigators in those studies, they may not reflect the actual time point at which the lumen diameter was stabilized. In addition, the variability in the simulation time course is constrained by the effects of using a default vessel thickness and assumed uniformity of the cell behaviors in the model.

All model parameters were based on values for human cells and cytokines reported in the literature. However, the exact regulation mechanism for many of these processes, including growth factor- and injury-regulated cell proliferation, has yet to be fully delineated. The necessary assumption was that the adopted model parameters held true for all cells of a given type in the simulated environment when, in fact, the behavior of cells *in vivo* is not uniform. Induced randomness in the model functions and using *in vivo* measurements for the vessel thickness would likely further increase the accuracy of the simulation. The use of different values for vessel wall thickness would impact simulation results since the subsequent alterations to the TGF-β release parameter is influenced by hoop stress, which is directly related to the vessel wall thickness and lumen radius (More details about hoop stress and its effect on TGF-β release rate can be found in Text S1).

The limitations of the model stem from the nature of computational modeling itself. The size constraints imposed on the model by the computing power of the NetLogo modeling environment negatively affected the scale the model could simulate by limiting the total number of agents that could be tracked within the simulation. While there are more powerful modeling environments that would partially alleviate this problem, attempting to simulate all the cells present in even a small cross-sectional area of a human blood vessel would be an unwieldy undertaking for any computational modeling environment.

In addition, it is difficult to recreate the precise natural variations seen in the behavior of cells and cytokines in the body. For instance, it is known that TGF-β has different behavioral patterns at different stages of disease development [38], and subsequently varies in its impacts on the behavior of other cells and cytokines. In the model, TGF-β was treated as a single-modality cytokine. One motivation for this type of simplification was that the properties of those cells and cytokines are not yet well-understood by investigators in the field. Thus, in order to recreate those variations seen *in vivo*, modelers would have to make many assumptions which might be random in nature and lack supporting evidence in the literature. The other motivation for the chosen simplification was that there are a large number of cell and cytokine types involved in the process of restenosis development. Attempting to include all of these cells and cytokines in the model would introduce a number of unknown or not-well-defined parameters into the model. Simplifications are a critical component in the construction of effective and rigorous models and can help investigators to concentrate on a limited number of essential components in the simulated system [55]. This approach may sacrifice the power of simulating the subtle differences of different cytokines but yield a much clearer understanding of the critical components that lead to in-stent restenosis.

However, the model created with these simplifications is fully capable of simulating the variations in cell and cytokine effects on a larger scale. As mentioned earlier in the Materials and Methods section, the visual outputs of multiple random model runs for simulations with the same set of input parameters produced different results. In some model simulation runs (roughly 40% of all runs), there was neointima layer thickening, but it did not reach the point of clinically-defined restenosis [51]. In other model runs, in-stent restenosis developed at different severity levels. This result reflects the variation seen in the clinical manifestation of in-stent restenosis. It is estimated that the rate of restenosis occurrence in patients who have undergone an angioplasty and stent procedure in randomized trials ranges between 30–40% [56], [57]. Other studies, however, have reported incidence rates as high as 48.8% [58] or even 60% [5]. This natural variation in restenosis rates as described in the literature was recreated by the model on both the tissue and organ scales.

There were some innate assumptions in the model construction process that merit discussion. For instance, it was assumed that the majority of cells in the neointimal layer were SMCs and their proliferation and migration were the driving forces of the renarrowing of the lumen, under the influence and regulation of multiple other cells and cytokines. This assumption was supported by the literature [59], although there are other known factors in this process, such as extracellular matrix deposition and vessel remodeling (or persistent change in vessel size) [60]. This model clearly indicated that SMC proliferation and migration is the most important driving force in the development of restenosis, as extracellular matrix deposition and vessel remodeling were ignored in the model, but the model still could simulate the development of restenosis with high accuracy. The model’s results do not signify that patients with higher SMC proliferation and migration will develop restenosis faster or more severely because of dynamic interactions among SMCs, other cells, and cytokines. From a clinical perspective, however, controlling SMC proliferation and migration may be a possible way to impede the development of restenosis. This is the therapeutic approach that drove the development of drug-eluting stents [59]. In that approach, the proliferation of SMCs and ECs is inhibited by the drug that coats on the drug-eluting stents. Unfortunately, such stents are not completely successful in improving patient outcomes because they are associated with other risks, such as late and very late thrombosis [61], [62].

As described previously in the Materials and Methods section, the model can be dynamically manipulated in the model interface by editing the values for parameters such as the vessel diameter, number of struts, size of struts, length of stent, space of struts, number of stents and stent alignment. Such manipulation has have already been demonstrated in the validation simulations with three sets of input parameters. This type of simulations is used to simulate a group of patients in a similar situation. Therefore variations are observed in those simulations. In the future, the authors plan to integrate more patient-specific parameters into the model interface, such as platelets, leucocytes, and cytokines counts; the size of the targeted plaque; and vessel wall thickness. The updated model would then be able to produce more patient-specific results.

The motivation for not including plaque rupture and the corresponding intense inflammatory reactions in this model were explained at the beginning of the Materials and Methods section. In the future, the role of plaque rupture could be taken into account in an extended model. In such a model, anti-platelet and anti-inflammatory drugs, lipid, and interferon-γ would be introduced as agents in the model. The size and location of plaque are also important in this case and therefore would have to be taken into account in the model as well. The ruptured plaque releases a large number of inflammatory factors in a short period of time and triggers the activation of a large number of platelets that subsequently results in acute thrombosis [18]. Without proper control, both the strong inflammatory reaction and the acute thrombosis may lead to patient death. Therefore, simulating the consequences of plaque rupture is a critically important research question.

As mentioned in the Introduction section, the vast majority of patients, approximately 92%, who received the PCI procedures also underwent stent procedures simultaneously [3], [63]. An important reason for this practice is that the elastic recoil right after balloon angioplasty can result in approximately 50% cross-sectional area loss on average [64]. Simultaneous stent placement is advantageous in that it can easily prevent elastic recoil from occurring. Conversely, implanting a stent also introduces further damage to the blood vessel. The model presented in this article was specifically created to simulate the response of the vessel to the concomitant angioplasty and stent procedures. However, since there are no similar models with a similar setting for the PCI procedure alone before this model, it can be problematic to determine the precise contributions from the stent in the development of in-stent restenosis. Accordingly, the result of this model cannot precisely identify the contributions of the stent to the lumen area loss when compared to the development of restenosis following a balloon angioplasty procedure. Therefore, even though the results obtained from the simulations in this model with varying stent parameters would be able to provide useful information for designing or testing different stents, a more accurate conclusion could be drawn after conducting simulations with a similar model where the consequence of angioplasty procedure alone is simulated. In the future, we plan to create an agent-based model to simulate the development of restenosis after only a balloon angioplasty procedure. The comparison between the results from these two models should be able to further clarify the contribution of a stent to restenosis development.

As mentioned earlier in this section, the drugs that coat on drug-eluting stents inhibit the proliferation of all types of cells in the blood vessel and therefore inhibit the development of in-stent restenosis. However, patients with a drug-eluting stent have to undergo prolonged anti-platelet drug therapy because the damage to the endothelial cell lining and the media layer during the angioplasty and stent procedure can never be recovered, making platelet activation and subsequent thrombus formation a constant risk [65]. Because drug-eluting stents can effectively help patients to avoid in-stent restenosis and there are cheap anti-platelet drugs, for instance, aspirin [66], they have been widely used in recent years [67]. However, anti-platelet drugs can become a heavy burden for patients who do not respond well to the typical pharmaceuticals and patients who need to take multiple types of anti-platelet drugs to effectively avoid thrombosis [68], [69].

In the future, we plan to create a model based on the setting of the current model that is capable of simulating the consequences of drug-eluting stent deployment. In that model, the proliferation and migration of SMC will not be so important while anti-platelet drugs will have an important role [70]–[73]. This drug-eluting stent model may be used to investigate the consequences of novel approaches and new technologies. For instance, the model would be helpful in investigating the possible results when the release of drug coated on the stent is delayed until the initial damage has been given time to heal. Such a controlled drug-eluting technology would be very promising because it would allow for inhibiting the proliferation of SMCs and ECs without the risk of late thrombosis. The model could provide critical information on the appropriate drug release timing to achieve this balance.

## Conclusions

The developed agent-based model represents a simulation of the interplay between the major contributors in the development of restenosis in a human atherosclerotic blood vessel after an angioplasty and bare-metal stent deployment. The model simulated the body’s response to the intervention and generated results consistent with serial imaging of human subjects across multiple studies. This model may serve as a tool to explore how different vessel cross-sections or stent arrangements may advance or reduce the development of restenosis in a blood vessel.

In the future, the authors will develop this model further to include more patient-specific physiological parameters and investigate the effects of plaque rupture. The authors will also create two parallel models, one for the angioplasty-only intervention and the other for the implantation of drug-eluting stents.

The model can be accessed at http://cpath.him.pitt.edu/stent/index.html.

## Supporting Information

Text S1 **Detailed explanation of local environment, behavioral rules, parameter selection, parameter sensitivity analysis, and simulation result consistency tests.**
(DOCX)
