# Supplementary material for: An Agent-Based Model of the Response to Angioplasty and Bare-Metal Stent Deployment in an Atherosclerotic Blood Vessel
Source: PLoS One. 2014 Apr 14;9(4):e94411. doi: 10.1371/journal.pone.0094411 (PMC3986389; doi:10.1371/journal.pone.0094411)
Supplement: Text S1 — Detailed explanation of local environment, behavioral rules, parameter selection, parameter sensitivity analysis, and simulation result consistency tests. (DOCX) [file pone.0094411.s001.docx]

**Local Environment**

In the agent-based model described in the main text, the local environment is defined as area of the patch where the agent of interest is located and the 8 immediately adjacent patches in the grid space. In Figure S1 below, the dark center patch contains the agent of interest, and the local environment encompasses the entire shaded area.

|  |  |  |  |  |  |  |  |  |
| --- | --- | --- | --- | --- | --- | --- | --- | --- |
|  |  |  |  |  |  |  |  |  |
|  |  |  |  |  |  |  |  |  |
|  |  |  |  |  |  |  |  |  |
|  |  |  |  |  |  |  |  |  |
|  |  |  |  |  |  |  |  |  |
|  |  |  |  |  |  |  |  |  |
|  |  |  |  |  |  |  |  |  |
|  |  |  |  |  |  |  |  |  |

**Figure S1. A local environment in the grid space.** The shaded region is a local environment of agents on the dark center patch, according to the definition of the NetLogo platform.

**Behavioral Rules of Agents in the Model**

A more detailed explanation of the agent-governing rules summarized in Table 1 is presented in the following paragraphs.

**SMCs**. The SMC agents are broken down into agent subtypes according to layer of the vessel wall in which they are located. These agent subtypes are associated with different rule sets. The SMC agent subtypes are named intimal SMCs, medial SMCs, and adventitial SMCs. Medial SMCs may migrate into the intimal layer during their proliferation [[1](#_ENREF_1),[2](#_ENREF_2)]. When a medial SMC agent crosses the medial-intimal boundary, it changes its agent type into an intimal SMC agent. These new intimal SMCs are indistinguishable from SMCs native to the intima and continue to proliferate in their new location, contributing to the progression of the neointima into the lumen [[2](#_ENREF_2)].

**ECs**. For both SMC and EC agents, local cytokine (TNF-α and TGF-β) concentrations are required to determine whether a particular SMC or EC agent should proliferate, undergo apoptosis, or neither. The specific rules and the relevant threshold concentrations are presented in the “Parameters” section below. To model the movement of the endothelial cell lining by the advancement of the neointima, endothelial cell agents always reside on patches adjacent and interior to the forward-most patches containing SMCs, and move accordingly in response to the advancement of intimal SMC agents.

**Platelets**. A series of checks is regularly performed on circulating platelets in the model. If the age of the platelet agent is over its lifespan threshold, that platelet agent will be killed. If the latent platelet makes contact with activated platelets or the wound site, that latent platelet agent will be converted into an activated platelet and fixed at its position at the time of activation. Activated platelets promote the release of TGF-β, thus contributing to the cytokine’s concentration in its local environment.

**Monocytes, Neutrophils and Macrophages**. When the local TGF-β concentration in the intimal layer exceeds the femtomolar level (10^-15^ moles per liter) [[3](#_ENREF_3)], circulating neutrophil and monocytes agents appear in the lumen. Neutrophil and monocyte agents are attracted to an injury site by a chemoattractant gradient of TGF-β. *In vivo,* this chemoattractant gradient is generated by the exposed subendothelial matrix [[3-6](#_ENREF_3)]. When circulating monocyte or neutrophil agents enter a patch containing an activated platelet or cross the lumen-thrombus boundary, the monocyte agents change into a migratory monocyte subtype and the neutrophils are integrated into the thrombus. Migratory monocytes then advance through the thrombus until they reach the tissue, where they change into one of two mature macrophage agent types: “alternately activated macrophages” (AAMs) or “classically activated macrophages” (CAMs). The AAMs remain localized around the **stent struts** and simulate the vessel’s foreign body to the struts [[7](#_ENREF_7)]. CAMs continue to migrate radially away from the lumen and trigger inflammatory reactions. When present, monocytes, neutrophils, and macrophage cells release TNF-α [[8](#_ENREF_8)].

**Parameters**

Further details about how appropriate values for the model’s parameters were chosen are provided below.

**TGF-β**. Activated platelets, ECs, medial SMCs, and intimal SMCs all release TGF-β at the same rate in the model. A measured *in vivo* release rate of 0.02 picogram/10 hours/cell (1 pictogram = 10^-15^ kg), was used as a baseline for the TGF-β release rate in the model [[9](#_ENREF_9)]. Intimal SMC agents in close proximity to stent struts release TGF-β at an increased rate, based on the observation that SMC proliferation is proportional to the extent of injury [[10](#_ENREF_10)], and the assumption that the underlying cause of this phenomenon is an increased release of TGF-β at sites of greater injury.

It is assumed that the persistence and rate of TGF-β release are constitutively related to the stress on the lumen wall. The calculation is loosely based on the formula for determining hoop stress on a cylinder [[11](#_ENREF_11)]. The formula for calculating hoop stress in a thin-walled cylinder is given below:

$$\sigma=\frac{P\times r}{t}$$

where σ is the hoop stress, *P* is the pressure on the cylinder wall, *r* is the internal radius of the cylinder, and *t* is the wall thickness of the cylinder. In the context of the model, the simulated blood vessel is treated as an approximate cylinder. As restenosis develops, the thickness of neointima increases while the radius of lumen decreases. If the neointima is considered as a *nested* cylinder (as demonstrated in Figure S2), one can use the same formula to calculate the hoop stress on this nested cylinder. The function for TGF-β release rate$C_{TGF-\beta}(\sigma)$is then defined as a ratio of these two hoop stresses:

$$C_{TGF-\beta}(\sigma)=\frac{\sigma_{n}}{\sigma_{i}}$$

Where $\sigma_{n}$is the hoop stress of the nested neointima-derived cylinder, and $\sigma_{i}$ is the initial hoop stress of the blood vessel. The initial post-stenting stress value is a constant in each simulation (determined by the initial vessel wall thickness and post-stenting lumen radius) while the stress of the nested cylinder changes as the thickness of neointima and the radius of lumen change. The effects of changes in blood pressure on the vessel wall were not integrated into the calculation. The TGF-β release rate is initially slightly higher than the rate measured in Facoetti et al. [[9](#_ENREF_9)], because of the existence of the high-releasing injured SMCs, and then decreases as the neointima becomes thicker. This formulation is based on the documented relationship between the degree of cell injury and the level of TGF-β release [[10](#_ENREF_10)], and the concept that the growth of the neointima represents the repair of the injury and likely corresponding to the reduction in TGF-β release.

TGF-β constantly diffuses into the patches surrounding the agent(s) that release it. There is a diffusion rate (13 μm^2^/s) of TGF- β measured *in vivo* [[12](#_ENREF_12)], however, this value is not directly applicable to the environment being modeled because of its scale. In this model, the diffusion rate was set such that it takes a TGF-β one day to diffuse through the vessel wall and into the lumen.


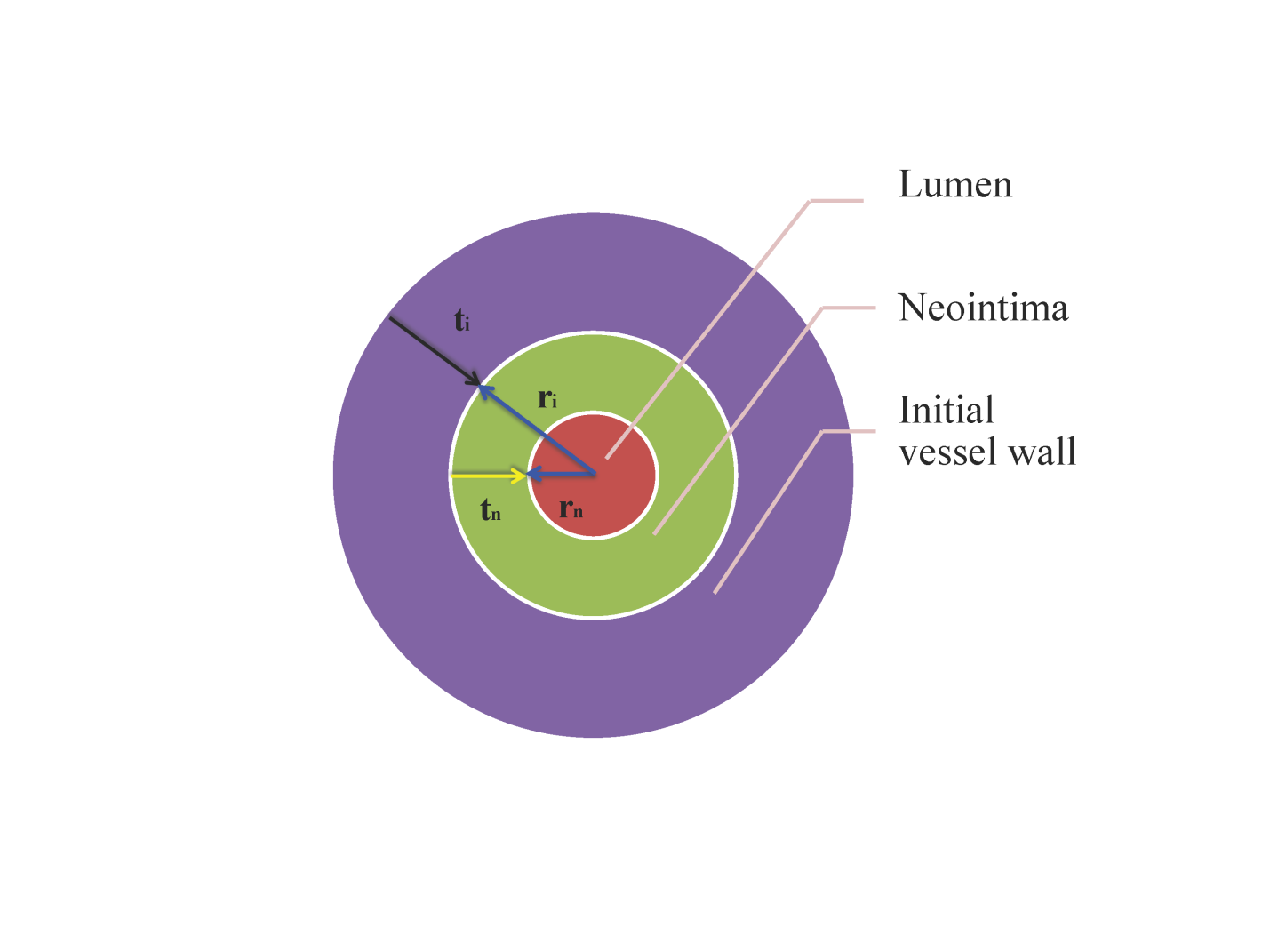


**Figure S2. Cylinder and its nested cylinder for hoop stress calculation.** The initial vessel wall following the stent procedure, the neointimal layer, and the final lumen area are indicated by labels. The wall thickness and the lumen radius for both the initial cylinder and its nested cylinder are illustrated as well. Here $t_{i}$ is the initial wall thickness of the cylinder, $r_{i}$ is the initial lumen radius of the cylinder, $t_{n}$ is the wall thickness of the nested cylinder, and $r_{n}$ is the final lumen radius of the nested cylinder.

**Monocytes, Neutrophils, and Macrophages**. The migration rate for monocyte, neutrophil and macrophage agents was based upon the typical *in vivo* migration rate of monocytes, which is approximately 1μm/ min [[13](#_ENREF_13)]. The *in vivo* lifespan of a macrophage is between1 and 3 months in the tissues, and the lifespan of a neutrophil is normally approximately 5 days, but in extreme cases may be as long as 12 days [[14](#_ENREF_14),[15](#_ENREF_15)]. Monocytes circulating in the blood can typically survive up to 3 days [[16](#_ENREF_16)]. Based on this data, the lifespans of macrophage, neutrophil, and monocyte agents in the model were set as 45 days, 5 days, and 3 days, respectively.

**TNF-α**. TNF-α can cause cell apoptosis, and cell apoptosis assays typically use TNF-α concentrations of 20 ng/ml (nanogram/milliliter or 10^-6^ kg/m^3^) although concentrations as low as 2.5 ng/ml have been shown to be sufficient to trigger apoptosis [[17](#_ENREF_17),[18](#_ENREF_18)]. More specifically, Rangamani et al. reported that a concentration of 2.5 ng/ml was required for initiating apoptosis, while Heikkilä *et al*. showed that approximately 15% apoptosis occurred with a concentration of 20 ng/ml [[17](#_ENREF_17),[18](#_ENREF_18)]. In this model, a local TNF-α threshold value of 4 ng/ml was selected, and the percentage of local apoptosis that occurs was scaled linearly such that roughly 15% of the local cells die if the local concentration of TNF-α reaches 20 ng/ml. These values were applied to EC agents as wells as to all subtypes of SMC agents.

Although the release rates of cytokines are likely to differ between cytokines types ­*in vivo,* the baseline release rates of TGF-β from literature were also applied to analogous situations with TNF-α, due to a lack of a firm values for TNF-α rates in the literature.

**SMCs and ECs**. The concentration of TGF-β in a local environment directs whether SMCs and ECs in that area should proliferate or not. The threshold concentration of TGF-β triggering SMC and EC proliferation is 3 ng/ml and selected based on a minimum concentration observed in patients with restenosis [[19](#_ENREF_19)]. Local concentration of TGF-β is evaluated in each layer of the vessel wall independently. If the local TGF-β concentration in one layer exceeds the threshold, the SMCs and/or ECs in that layer replicate at a rate such that their population doubles every 30 hours. This rate is based on a reported human EC proliferation rate measured *in vitro* [[20](#_ENREF_20)]. The SMCs that are designated as “injured intimal SMCs,” based on their proximity to the stent struts, replicate twice during every proliferative cycle [[10](#_ENREF_10)]. Proliferating medial SMC agents migrate at a rate of 20 μm/hour, which is the rate of human SMC migration across a fibronectin and collagen substrate [[21](#_ENREF_21)].

**Plaque**. To indicate the size and location of the plaque, four coordinates (x_1_, x_2_, y_1_, y_2_) were used to define the boundaries of the plaque area. As described at the beginning of the Methods section, it was assumed that the plaque neither ruptures nor protrudes into the lumen in this model. It is additionally assumed that the struts of stent did not impale the plaque during implant. Lastly, it is also assumed that the shape of the blood vessel cross-section is a circle after the angioplasty and stent procedure. As a result of these assumptions, the plaque does not contribute to the inflammatory reaction, thrombus formation, or the restenosis development.

**Parameter Sensitivity Analysis**

Although the model is made up of many functions and parameters, they are not of equal consequence. The two functions with the most significant impact on the results of the model are not directly related to physiological parameters. The first such functions is the scaling of the relationship between $\sigma_{n}$ and $\sigma_{i}$ in the TGF-β release rate equation. Those coefficients in this function dictate the time to lumen diameter stabilization following the stent deployment, as well as the final lumen diameter. The final values for the coefficients that govern the reduction of the TGF-β release rate based on $\sigma_{n}$ and $\sigma_{i}$ were chosen by performing a rigorous series of experimental simulations. Once selected, these values were not altered during any subsequent simulations, regardless of model input parameters. As noted previously, the baseline release rate itself was based on the literature [[9](#_ENREF_9)].

The second crucial effector of the model is the limit on the number of SMC agents per patch in the grid. According to the behavioral rule, once the number of SMCs on one patch goes beyond the given limit (the number is 15 in the model), one SMC agent on that patch will be pushed onto an adjacent patch in the direction of the lumen. This displacement advances the neointimal vessel wall boundary toward the lumen. Thus, the SMC per patch limit dictates the speed at which the simulation reaches a stable lumen diameter. Similar to the approach for determining the coefficients of $\sigma_{n}$ and $\sigma_{i}$, the appropriate limit for the number of SMCs per patch was identified by performing a series of experimental simulations, and the chosen value was subsequently unaltered during the validation simulations and the overlapping-stent simulations.

Since the maximum number of SMCs on one patch is critical to the speed of developing the restenosis, the lumen diameter stabilization time, and the final lumen size, altering this parameter can modulate the rate and severity of restenosis developed by the simulated patient. If this SMC per patch limit represents a physiological parameter, it would mean that a crucial factor in the restenosis development has been identified. Unfortunately the authors are not aware at this time of any relevant physiological parameter that corresponds to this model parameter.

After the appropriate parameter values for the two critical functions described above were determined, a few hundred simulations were performed to observe the consistency of the simulation results. The results are provided in Table S1 and S2. The reported results in these two tables were from 10 random runs with each of the indicated alterations applied. For each given set of initial parameters, the final lumen diameters obtained from those simulations were consistent.

**Result Consistency Tests**

One simple test was conducted using one set of input parameters to demonstrate this consistency. In this test, the initial lumen diameter was 2.9 mm and the simulation was run 30 times. The final lumen diameters were collected. Two groups of these results (10 results in each group) were randomly selected without replacement. The values of final lumen diameter in the first group were 1.63, 2.15, 0.42, 2.13, 1.31, 2.13, 1.87, 1.97, 1.88, and 2.15. The values of the final lumen diameter in the second group were 2.15, 1.70, 1.85, 1.73, 1.83, 2.13, 1.86, 0.56, 1.83, and 1.86. The average of these two groups of final lumen diameters were 1.764 and 1.750, respectively. The average of final lumen diameters from these 30 runs was 1.765. A Welch two sample *t-* test was performed on these two independent random samples to find out whether the difference of these two average values is statistically significant. The obtained p-value from this *t*-test was 0.9504. The null hypothesis (the averages of the two samples are the same) is accepted. In other words, the average values of the two groups with 10 random runs each are highly similar or consistent since there is no statistically significant difference between them. One sample Student’s *t*-tests were also performed on these two samples against the population average (1.765) and the obtained p-values were 0.9955 and 0.9172, respectively. These results further confirmed that it is sufficient to have 10 runs per simulation for data reporting. Therefore, in the results section of the main text, all of the reported results are the average values of 10 random runs of the simulation with the indicated parameters.

**Table S1.** Results of the cytokine-based sensitivity analysis testing.

| **Agent Type** | **Function** | **Alteration Type** | **Effect of Alteration on Simulations** | **Explanation in the Context of the Model Organization** |
| --- | --- | --- | --- | --- |
| TNF-α | TNF-α migration rate | Double rate | 325% increase in rate of lumen diameter stabilization | TNF-α passes through local environment too quickly and the local threshold required to trigger apoptosis is achieved in fewer areas |
|  |  | Halve rate | 78% increase in rate of lumen diameter stabilization | Reduced area of apoptosis due to slowed TNF-α migration increases speed with which vessel wall expands |
|  |  | Turn function off | 20% increase in rate of lumen diameter stabilization | Increased local apoptosis levels partially counter-balances effects of reduced area of apoptosis |
|  | TNF-α release rate | Double rate | No effect | Increased TNF-α causes super-saturation of the local environment, but does not cause further apoptosis, as the threshold for maximum apoptosis is already reached at points within the model at the normal TNF-α rate |
|  |  | Halve rate | 260% increase in rate of lumen diameter stabilization | Reduced apoptosis from reduced local levels of TNF-α increases speed with which vessel wall expands |
|  |  | Turn function off | 400% increase in rate of lumen diameter stabilization | Reduced apoptosis from lack of TNF-α increases speed with which vessel wall expands |
| TGF-β | TGF-β migration rate | Double rate | 60% decrease in rate of lumen diameter stabilization | TGF-β passes through local environment too quickly and the local threshold required to trigger proliferation is achieved in fewer areas |
|  |  | Halve rate | No effect | Increased local proliferation levels completely counter-balance effects of reduced area of proliferation |
|  |  | Turn function off | 13% decrease in rate of lumen diameter stabilization | Increased local proliferation levels partially counter-balance effects of reduced area of proliferation |
|  | TGF-β release rate | Double rate | 60% increase in rate of lumen diameter stabilization | Increased local TGF-β concentrations result in more areas above the local threshold required to trigger proliferation, increasing the speed with which vessel wall expands |
|  |  | Halve rate | 16% decrease in rate of lumen diameter stabilization | Decreased local TGF-β concentrations result in fewer areas above the local threshold required to trigger proliferation |
|  |  | Turn function off | Model simulations run indefinitely | Insufficient pro-proliferative chemokines are present to stimulate proliferation, and lumen diameter is never stabilized |

**Table S2.** Results of the cell-based sensitivity analysis testing.

| **Agent Type** | **Function(s)** | **Alteration Type** | **Effect of Alteration on Simulations** | **Explanation Based on Model Organization** |
| --- | --- | --- | --- | --- |
| Platelets | All | Any | No effect | Platelets do not contribute significantly to change in lumen diameter and are not the sole cell types releasing any cytokine. |
| ECs | Border advancement | Turn function off | Model simulations run indefinitely | Model uses interior-most band of endothelial cells in order to measure lumen diameter; without endothelial cell advancement model detects no change in lumen diameter and lumen diameter never stabilizes |
|  | Proliferation | Turn function off | Model Error | Density of endothelial cells becomes too sparse for model to measure change in lumen diameter accurately |
|  | Apoptosis | Turn function off | No effect | Increased endothelial cell density does not contribute to lumen wall thickness, and does not affect lumen diameter measurement accuracy |
| SMCs | Adventitial proliferation and apoptosis | Any | None | Adventitial SMC population does not contribute significantly to vessel wall expansion |
|  | Intimal proliferation | Turn function off | 50% decrease in rate of lumen diameter stabilization | Halting intimal proliferation affects not only those SMCs native to the neointima, but also those that migrate from the medial layer. As a result, the speed with which the vessel wall expands is slowed. |
|  | Intimal apoptosis | Turn function off | 250% increase in rate of lumen diameter stabilization | Eliminating intimal apoptosis removes the check on intimal proliferation and allows for faster vessel wall expansion |
|  | Medial proliferation | Turn function off | 20% decrease in rate of lumen diameter stabilization | Halting medial proliferation also reduces the number of medial SMCs available to migrate and contribute to the intimal SMC population. |
|  | Medial apoptosis | Turn function off | 360% increase in rate of lumen diameter stabilization | Eliminating medial apoptosis removes the check on medial proliferation, providing more medial SMCs to migrate into the intima, and allows for faster vessel wall expansion |
| Circulating neutrophils and monocytes | Adherence | Turn function off | 340% increase in rate of lumen diameter stabilization | By preventing neutrophil and monocyte adherence, these leukocyte types will never enter the vessel wall and thus the population of TNF-α-releasing cells will be insignificant |
| Macrophages and monocytes in vessel wall | Migration | Turn function off | 300% increase in rate of lumen diameter stabilization | By preventing macrophage and monocyte migration, the radial permeation of TNF-α through the vessel wall will be reduced |

**References**

1. Roger VL, Go AS, Lloyd-Jones DM, Benjamin EJ, Berry JD, et al. (2012) Heart disease and stroke statistics--2012 update: a report from the American Heart Association. Circulation 125: e2-e220.

2. Newby AC, Zaltsman AB (2000) Molecular mechanisms in intimal hyperplasia. J Pathol 190: 300-309.

3. Ashcroft GS (1999) Bidirectional regulation of macrophage function by TGF-beta. Microbes Infect 1: 1275-1282.

4. Cerletti C, Tamburrelli C, Izzi B, Gianfagna F, de Gaetano G (2012) Platelet-leukocyte interactions in thrombosis. Thromb Res 129: 263-266.

5. Seye CI, Kong Q, Yu N, Gonzalez FA, Erb L, et al. (2007) P2 receptors in atherosclerosis and postangioplasty restenosis. Purinergic Signal 3: 153-162.

6. Stahl AL, Sartz L, Nelsson A, Bekassy ZD, Karpman D (2009) Shiga toxin and lipopolysaccharide induce platelet-leukocyte aggregates and tissue factor release, a thrombotic mechanism in hemolytic uremic syndrome. PLoS ONE 4: e6990.

7. Anderson JM, Rodriguez A, Chang DT (2008) Foreign body reaction to biomaterials. Semin Immunol 20: 86-100.

8. Monraats PS, Pires NM, Schepers A, Agema WR, Boesten LS, et al. (2005) Tumor necrosis factor-alpha plays an important role in restenosis development. FASEB J 19: 1998-2004.

9. Facoetti A, Mariotti L, Ballarini F, Bertolotti A, Nano R, et al. (2009) Experimental and theoretical analysis of cytokine release for the study of radiation-induced bystander effect. Int J Radiat Biol 85: 690-699.

10. Duckers H, J., Nabel EG, Serruys PW (2007) Essentials of restenosis: For the interventional cardiologist. Totowa, NJ: Humana Press.

11. Budynas RG, Nisbett J (2011) Shigley's mechanical engineering design. New York: McGraw-Hill.

12. Brown DR (1999) Dependence of neurones on astrocytes in a coculture system renders neurones sensitive to transforming growth factor beta1-induced glutamate toxicity. J Neurochem 72: 943-953.

13. Noma H, Kato T, Fujita H, Kitagawa M, Yamano T, et al. (2009) Calpain inhibition induces activation of the distinct signalling pathways and cell migration in human monocytes. Immunology 128: e487-496.

14. Heidenreich S (1999) Monocyte CD14: a multifunctional receptor engaged in apoptosis from both sides. J Leukoc Biol 65: 737-743.

15. Pillay J, den Braber I, Vrisekoop N, Kwast LM, de Boer RJ, et al. (2010) In vivo labeling with 2H2O reveals a human neutrophil lifespan of 5.4 days. Blood 116: 625-627.

16. Ferkol T, Perales JC, Mularo F, Hanson RW (1996) Receptor-mediated gene transfer into macrophages. Proc Natl Acad Sci USA 93: 101-105.

17. Rangamani P, Sirovich L (2007) Survival and apoptotic pathways initiated by TNF-alpha: modeling and predictions. Biotechnol Bioeng 97: 1216-1229.

18. Heikkila HM, Latti S, Leskinen MJ, Hakala JK, Kovanen PT, et al. (2008) Activated mast cells induce endothelial cell apoptosis by a combined action of chymase and tumor necrosis factor-alpha. Arterioscler Thromb Vasc Biol 28: 309-314.

19. Wildgruber M, Weiss W, Berger H, Wolf O, Eckstein HH, et al. (2007) Association of circulating transforming growth factor beta, tumor necrosis factor alpha and basic fibroblast growth factor with restenosis after transluminal angioplasty. Eur J Vasc Endovasc Surg 34: 35-43.

20. Sagnella SM, Kligman F, Anderson EH, King JE, Murugesan G, et al. (2004) Human microvascular endothelial cell growth and migration on biomimetic surfactant polymers. Biomaterials 25: 1249-1259.

21. DiMilla PA, Stone JA, Quinn JA, Albelda SM, Lauffenburger DA (1993) Maximal migration of human smooth muscle cells on fibronectin and type IV collagen occurs at an intermediate attachment strength. J Cell Biol 122: 729-737.
